# Supplementary material for: A combined experimental-computational approach uncovers a role for the Golgi matrix protein Giantin in breast cancer progression
Source: PLoS Comput Biol. 2023 Apr 17;19(4):e1010995. doi: 10.1371/journal.pcbi.1010995 (PMC10159355; doi:10.1371/journal.pcbi.1010995)
Supplement: S1 Text — Fig A. Impact of cell speed and persistence on tumor progression. (A) Strong negative correlation between number of necrotic cells and uniformity index (Pearson correlation coefficient = -0.76). (B) Weak negative correlation between number of quiescent cells and uniformity index (Pearson correlation coefficient = -0.24). (C) Number of cancer cells after 90 days from the tumor initiation across different speed-persistence combinations. (D) Number of cancer cells at different time points (blue: day 70, yellow: day 80 and gray: day 90) across gradual persistence value but fixed speed of 30um/h. (E) Number of intravasating cells after 90 days from the tumor initiation across different speed-persistence combinations. Error bars represent the standard deviation of twenty five replicates. Fig B. Immunofluorescence staining for Golgi marker giantin (yellow). (A) Representative image of adjacent normal breast tissue patient samples. (B) Representative image of invasive ductal carcinoma patient section. Fig C. Kaplan–Meier survival curves for six matrix proteins that have a relatively well understood role in maintaining Golgi morphology (GOLGA1, GOLGA2, GOLGA3, GRASP55, GOLGB1 and GORASP1). Fig D. Impact of Golgi fragmentation on cell migration. (A) Percentage of cells denoted with fragmented Golgi in BT549 cells. (B) Bar plot showing the percentage of cells based on speed, low speed includes group 1&2 whereas high speed includes group 3&4. (C) Bar plot showing the percentage of cells based on persistence, low persistence includes group 1&3 whereas high persistence includes group 2&4. (D) Bar plot showing the averaged persistence ratio per group. Fig E. Number of intravasating cells in a population of a thousand cancer cells. Statistical significance was determined using the Kruskal–Wallis rank sum test, followed by pairwise comparisons using the Wilcoxon rank sum test with Bonferroni Correction (* p < 0.05, *** p < 0.001). Fig F. Cell’s speed-persistence. (A) 5-day speed pro [file pcbi.1010995.s001.docx]

**A combined experimental-computational** **approach uncovers a role for the Golgi matrix protein Giantin in breast cancer progression**

Salim Ghannoum^1ŧ*^, Damiano Fantini^2ŧ^, Muhammad Zahoor^1^, Veronika Reiterer^3^, Santosh Phuyal^1^, Waldir Leoncio Netto^4^, Øystein Sørensen^5^, Arvind Iyer^6^, Debarka Sengupta^7,8^, Lina Prasmickaite^9^, Gunhild Mari Mælandsmo^9,10^, Alvaro Köhn-Luque^4*^, Hesso Farhan^1,3*^

^1^ Institute of Basic Medical Sciences, Department of Molecular Medicine, University of Oslo, Oslo, Norway.

^2^ Department of Urology, Northwestern University, Chicago, Illinois, USA.

^3^ Institute of Pathophysiology, Medical University of Innsbruck, Innsbruck, Austria

^4^ Oslo Centre for Biostatistics and Epidemiology, Faculty of Medicine, University of Oslo, Oslo, Norway.

^5^ Center for Lifespan Changes in Brain and Cognition, Department of Psychology, University of Oslo, Oslo, Norway.

^6^ Department of Computational Biology, University of Lausanne (UNIL), Lausanne, Switzerland.

^7^ Department of Computational Biology, Indraprastha Institute of Information Technology, New Delhi, India.

^8^ Centre for Artificial Intelligence, Indraprastha Institute of Information Technology, Delhi, India.

^9^ Department of Tumor Biology, Institute for Cancer Research, Oslo University Hospital, The Norwegian Radium Hospital, Oslo, Norway.

^10^ Department of Medical Biology, UiT - The Arctic University of Norway, Tromsø, Norway

^ŧ^ Shared first-authorship

^*^ salim.ghannoum@medisin.uio.no (SG); alvaro.kohn-luque@medisin.uio.no (AKL); hesso.farhan@i-med.ac.at (HF)

D.F. is an employee of Xilio Therapeutics and a former employee and a shareholder of Eli Lilly and Company. The other authors declare that they have no competing interests.

**Fig A**


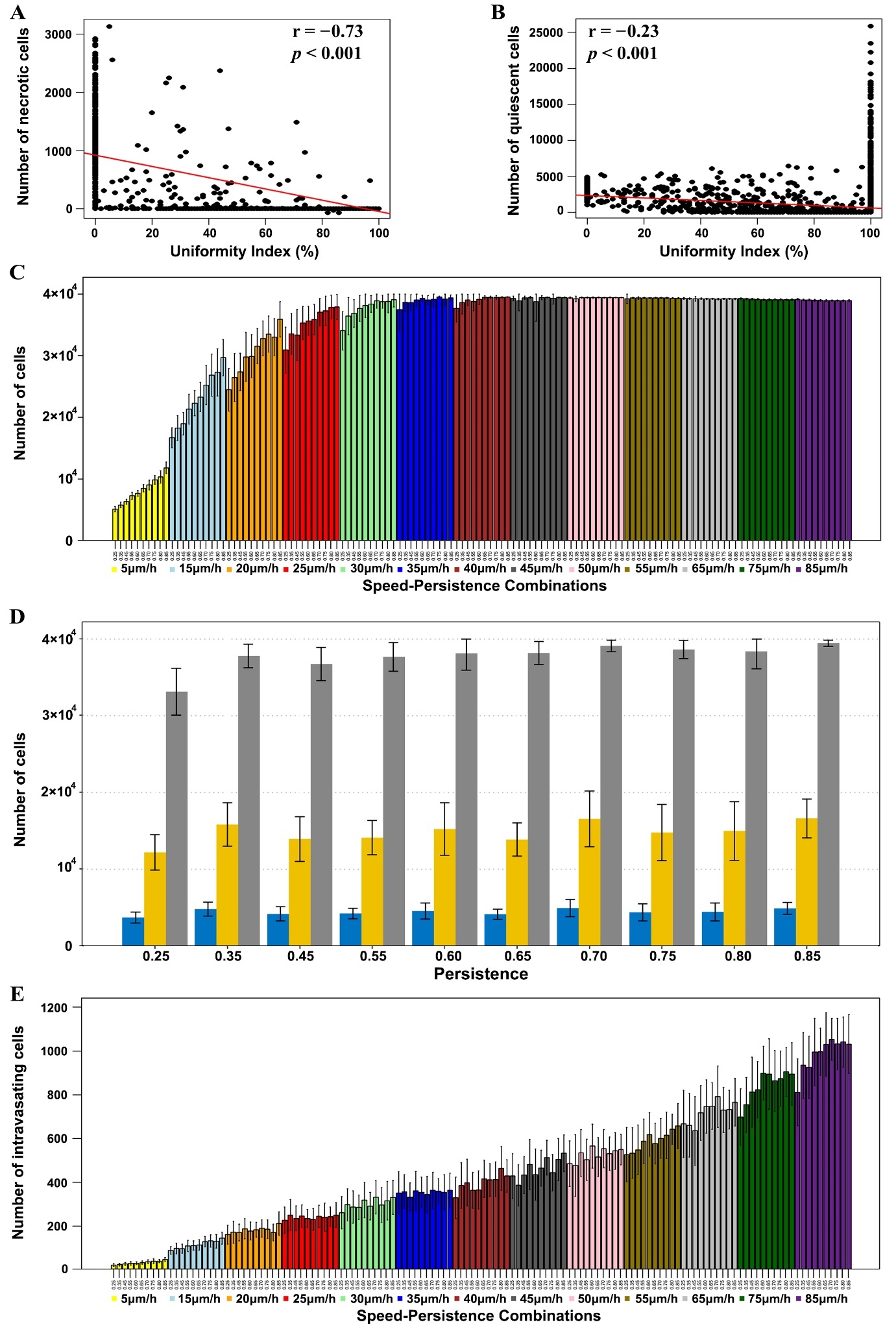


***Fig A. Impact of cell speed and persistence on tumor progression.*** *(A) Strong negative correlation between number of necrotic cells and uniformity index (Pearson correlation coefficient = -0.76). (B)  Weak negative correlation between number of* quiescent *cells and uniformity index (Pearson correlation coefficient = -0.24). (C) Number of cancer cells after 90 days from the tumor initiation across different speed-persistence combinations. (D) Number of cancer cells at different time points (blue: day 70, yellow: day 80 and gray: day 90) across gradual persistence value but fixed speed of 30um/h. (E) Number of intravasating cells after 90 days from the tumor initiation across different speed-persistence combinations. E*rror bars represent *the* standard deviation *of twenty five replicates.*

**Fig B**

**
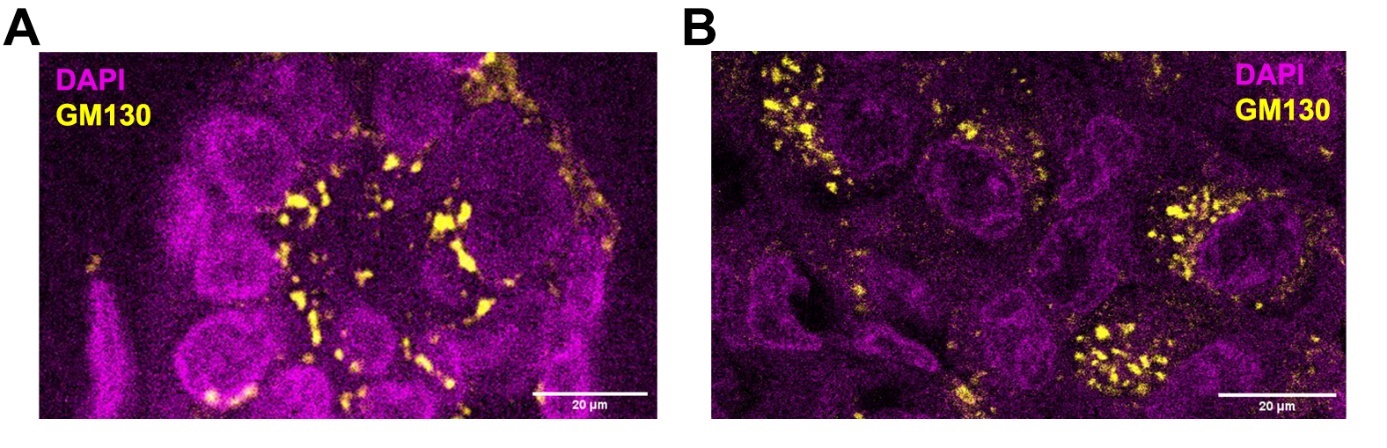
**

***Fig B. Immunofluorescence staining for Golgi marker giantin (yellow).*** *(A) Representative image of adjacent normal breast tissue patient samples. (B) Representative image of invasive ductal carcinoma patient section.*

**Fig C**

**
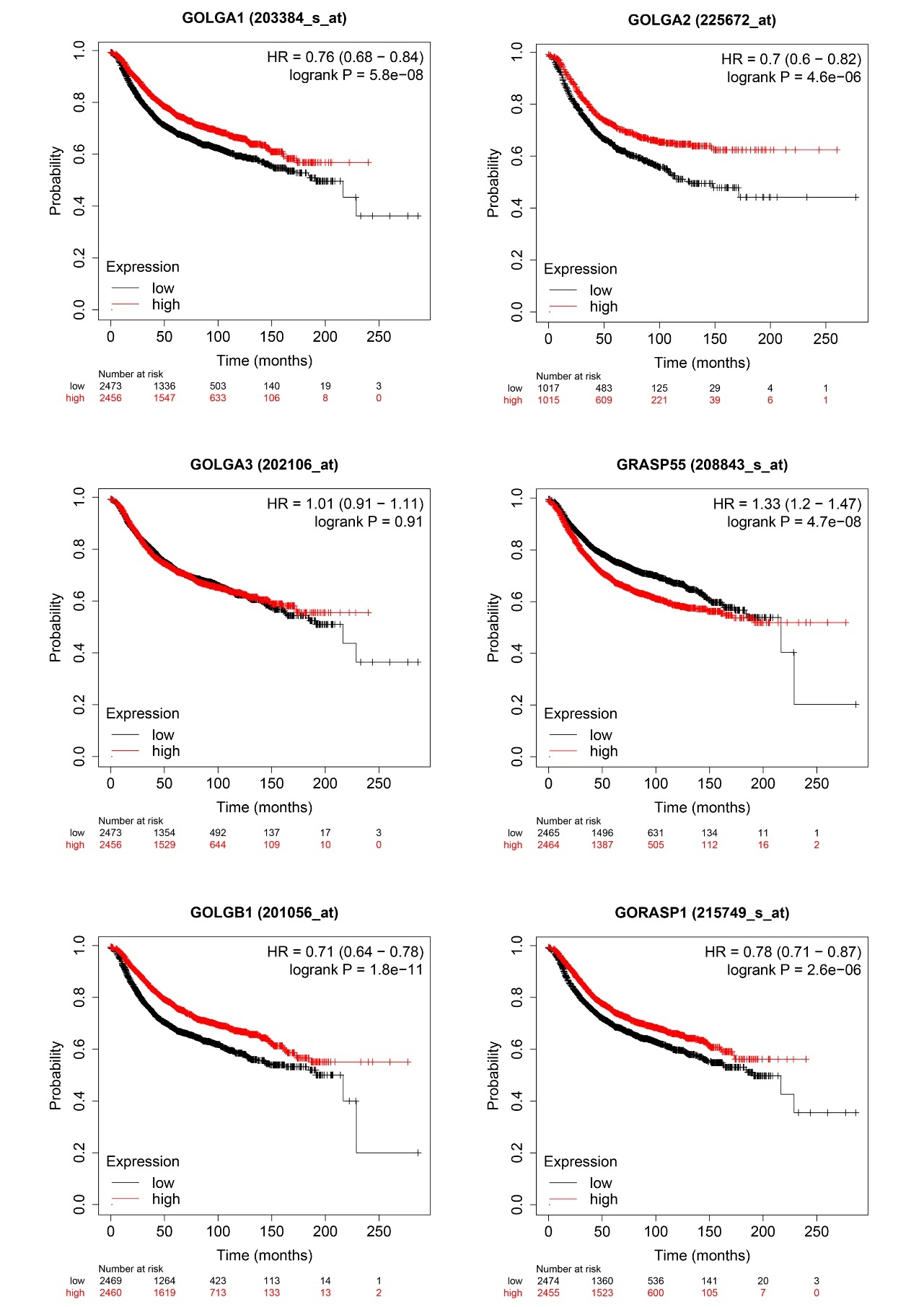
**

***Fig C. Kaplan–Meier survival curves*** *for* six matrix proteins that have a relatively well understood role in maintaining Golgi morphology (*GOLGA1, GOLGA2, GOLGA3, GRASP55, GOLGB1* and *GORASP1*).

**Fig D**


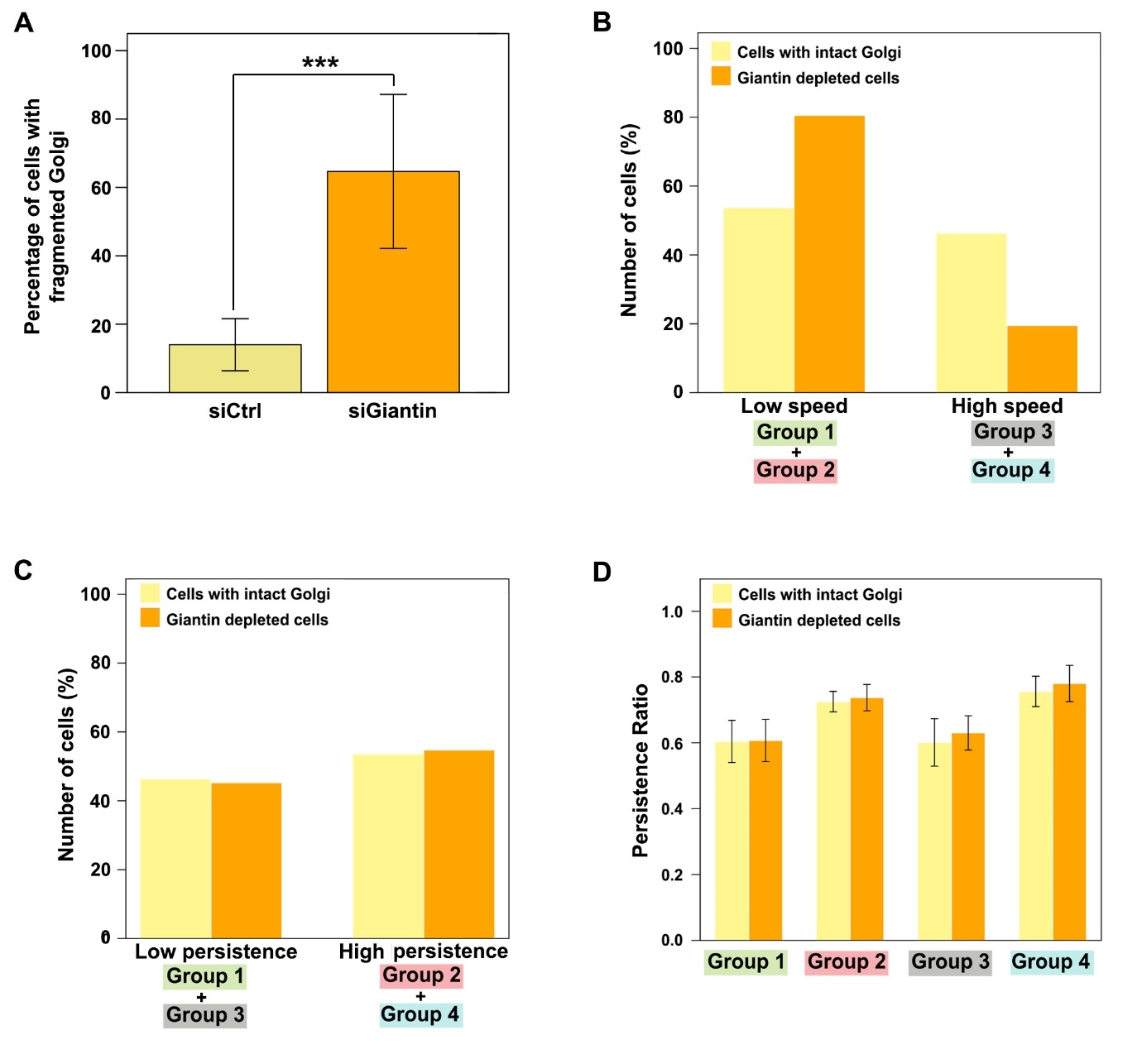


***Fig D. Impact of Golgi fragmentation on cell migration.*** *(A) Percentage of cells denoted with fragmented Golgi in BT549 cells. (B) Bar plot showing the percentage of cells based on speed, low speed includes group 1&2 whereas high speed includes group 3&4 . (C) Bar plot showing the percentage of cells based on persistence, low persistence includes group 1&3 whereas high persistence includes group 2&4. (D) Bar plot showing the averaged persistence ratio per group*

**Fig E**

**
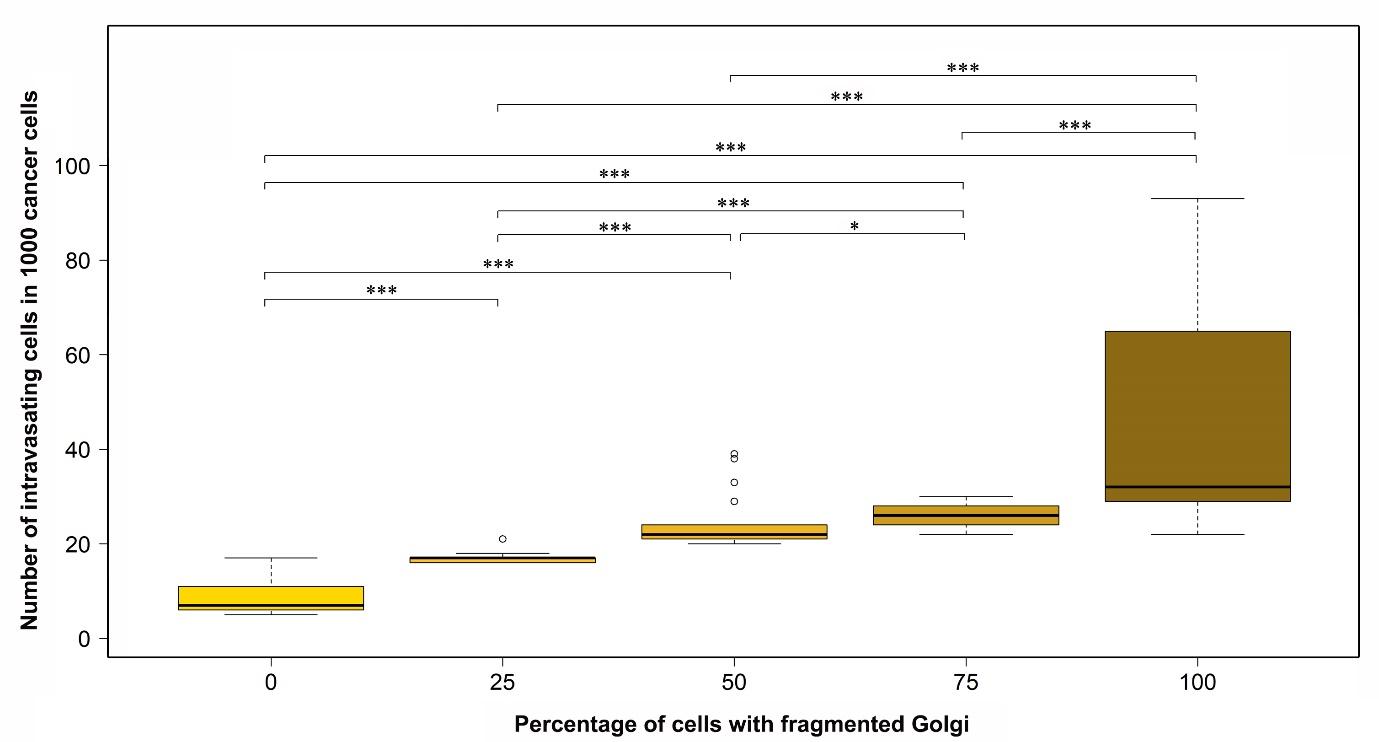
**

***Fig E. Number of intravasating cells in a population of a thousand cancer cells.*** *Statistical significance was determined using the Kruskal–Wallis rank sum test, followed by pairwise comparisons using the Wilcoxon rank sum test with* Bonferroni Correction *(* p < 0.05, *** p < 0.001).*

**Computational model of breast cancer growth and progression**

We use a stochastic cell-based model, built on the persistence random walk paradigm, to describe not only cell migration but also proliferation, quiescence, intravasation and cell death. This section describes in full detail: 1) the model assumptions; 2) how those assumptions are translated into a mathematical and computational framework; 3) the used model parameters and initial conditions; and 4) all computational details needed to simulate the model.

1. **Model assumptions**

Our stochastic model is based on 8 main assumptions:

i) Cells are individual active agents that, for simplicity, live in a 2D section.

ii) Cell movement is characterized by a given speed and directionality.

iii) The 2D tissue section receives nourishment from functional blood vessels that are assumed to be perpendicular to the tissue section.

iv) Cells in the nourished zone adjacent to a functional blood vessel are more likely to proliferate faster and move within this zone than leave it.

v) Besides proximity to blood vessels, cell divisions are also influenced by the local cell density and the time since the division of the corresponding mother cell.

vi) Cells that spend the G1 phase in the nourished zones can have a shorter cell cycle length.

vii) Cells become necrotic and die if they are not able to divide due to lack of space after a number of cell cycles. Quiescent cells are also considered. Those are cells that are in a transition state between a proliferating state and a total cell cycle arrest. They can shift back to proliferating state if the local cell density changes.

viii) Cells moving towards blood vessels can intravasate.

1. **Model description**

Here we translate the model assumptions into a continuous space (2D) and discrete time mathematical and computational framework:

i) Single cells are described as circular discrete agents with a certain diameter (*d*). The position of each agent is determined by the coordinates of its center that can be at any point in a continuous 2D simulation field (*F*). At any given time point, two agents cannot overlap, but to move from one location to another in each time step, an agent can cross through locations that are occupied by other agents.

ii) Agents change their coordinates across discrete time steps by a given speed and directionality. Instead of having constant speed, the agents follow speed distributions computed empirically (Fig F, panel A). Cells’ directionality is determined in term of persistence ratio (*P_r_*) which is computed as:

*P_r_ = N_p_/(N_p_ + N_d_)*                                                         (Equation S1)

where, *N_p_* is the number of persistent steps and N_d_ is the number of deviating steps (Fig F, panel B). Time-step movement vectors, referred to as steps, are classified either persistent or deviating based on the angular degree between the extension of the previous movement distance and the current one (Fig F, panel B). Persistent steps are associated with acute angles (*< π/2*) whereas deviating steps are associated with obtuse angles (*≥ π/2*).

iii) For simplicity, cross sections of blood vessels (*L_i_, i=1…N_V_*), referred to as lumina, are stochastically distributed in the simulation field (*F*) and modelled by squares with fixed area (*L*).

*
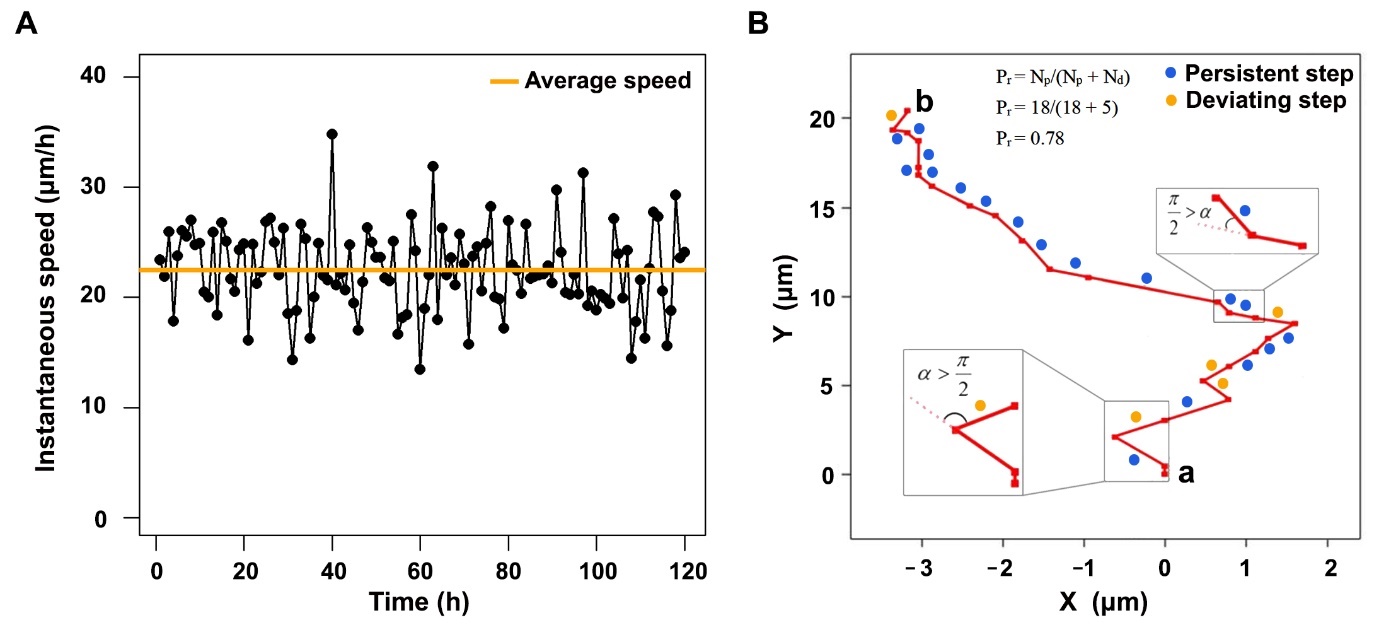
*

***Fig F. Cell’s speed-persistence.*** *(A) 5-day speed profile. (B) Schematic chart explaining how the persistence ratio is computed for a cell that moved from a to b.*

iv) Spatial zones around lumina (*W_i,_ i=1…N_V_* ), referred to as well-nourished zones, are also modelled as squares of area *W*, with *W > L*, surrounding the lumina (Fig G, panel A). When the position of an agent enters these zones, it has a higher probability to remain there than leaving to the poorly-nourished zone (*w*), which is defined as the space in the computational domain F that is not occupied by the union of the Nv well-nourished zones:

*w = F \ U_i=1..Nv_W_i_*                                         (Equation S2)

v) Cell division is a stochastic event. The probability of an agent to divide (*P_d_*) is based on a proliferation score (*PS*) that depends on 3 additive factors: the time since the division of the original agent (*T*), the local cell density (*D*) and proximity to a blood vessel (*V*). At each time point, these three factors are monitored for every cell in the simulated field. PS is computed as:

*PS = T +D+ V*                                               (Equation S3)

The local cell density (*D*) is a discrete index reflecting the number *n=0,1,..,8* of occupied spots around the target simulated cell in Moore neighborhood, regardless of the location of the occupied spots (Fig G, panel B). The index *D* is positive for low neighborhood occupancy (*n=0, 1, 2, 3*), zero for medium occupancy (*n=4,5*) and negative for high occupancy (*n=6,7,8*). However, for *n=8* we will assume proliferation is fully inhibited.

The proximity to a blood vessel (*V*) describes the proliferation advantage that a cell can get based on its location. V is computed as:

*V = B          (if Z = 1)*                                          (Equation S4)

*V = 0          (if Z = 0)*                                          (Equation S5)

where, *Z* refers to the location of the target cell in a poorly nourished zone *w* (*Z = 0*) or in a well-nourished zone *w^c^* (*Z = 1*), which is an advantage. This advantage is simulated as an additive bounce (*B*) that is equal to the time difference between the needed time for a cell to divide in a well-nourished zone (d_tw_) and a poorly nourished zone (*d_t_*):

*B =  d_t_ -  d_tw_*                                           (Equation S6)

We then assume a high division probability (*h*) when the proliferation score (*PS*) is equal or higher than the duration of the entire cell cycle (*d_t_*). Instead, we assume a very low division probability (*l*) when the proliferation score (*PS*) is lower than the cell cycle duration (*d_t_*):

*P_d_ = l           (if PS < d_t_)*                                          (Equation S7)

*P_d_ = h              (if PS ≥ d_t_)*                                          (Equation S8)

When a division takes place, two new daughter agents are generated. One agent keeps the position of the original agent. The second agent is placed stochastically based on the available spots in the Moore neighborhood around the original agent before it divided. The further movements of each of the two daughter cells are computed based on given speed and directionality. Each of the daughter cells moves from its initial location to a new location by a distance computed based on the given speed. The direction of the movement is determined based on the given directionality. Putting together the computed distance and directionality, the location of the new movement can be any free point on the semicircle-circumference with a radius of the computed distance (Fig H). Points occupied by other cells are excluded before stochastically selecting the location of the new movement.


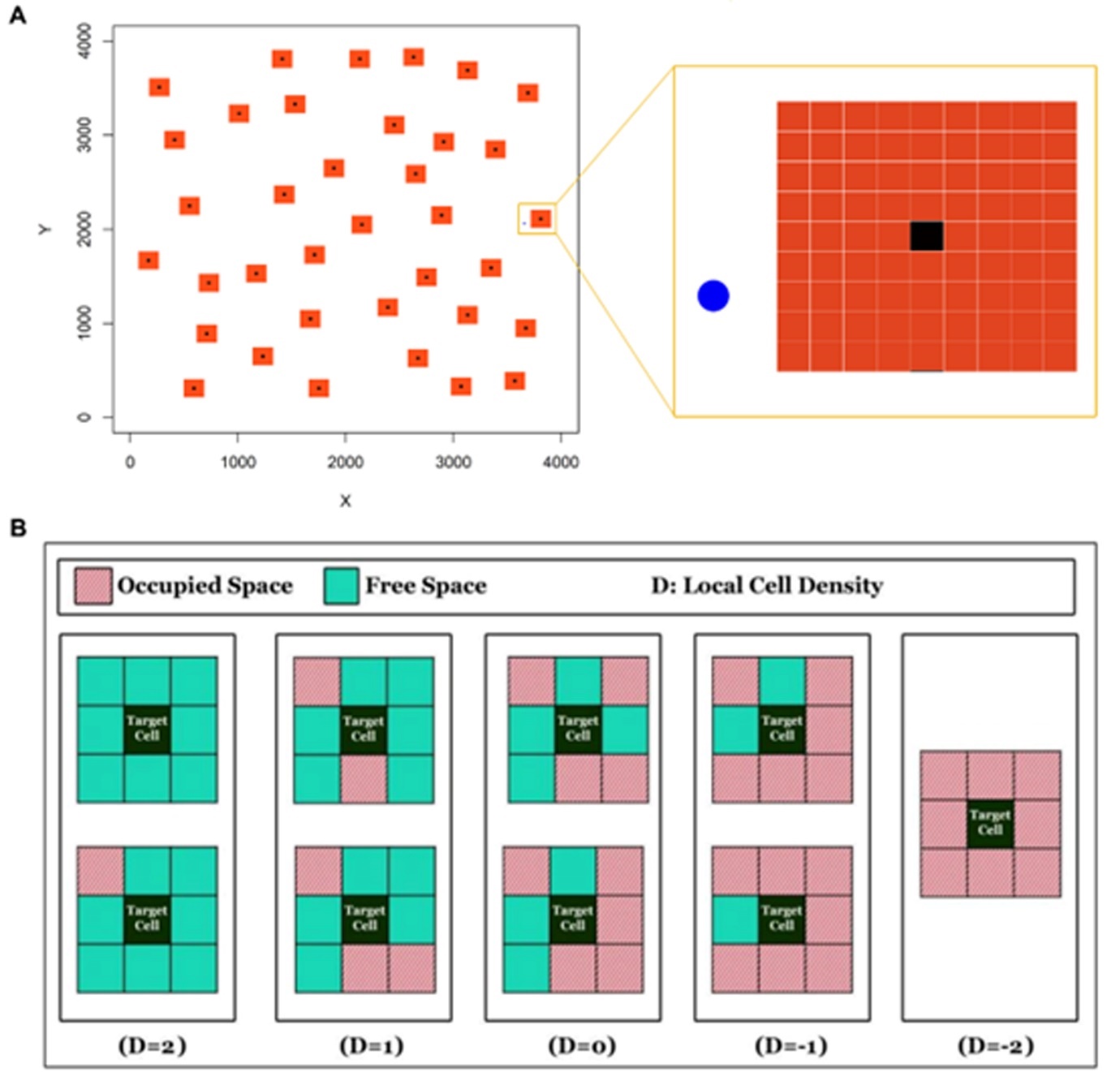


***Fig G. Simulation field and the local density.*** *(A) A 2D section showing the architecture of the simulated tumor field. Black squares represent the lumen of blood vessels. Red squares represent the well-nourished zones. The blue circle represents a single cancer cell. (B) Schema explaining the way of computing the local cell density.*

vi) We assume that after each cell division, the two cells start the first phase of the cell cycle  known as G1 phase. If a cell spends the needed time to complete the G1 phase (*t_G1_*) in a well-nourished zone (*Z = 1*) then it switches to high division probability, regardless of the value of *PS*.


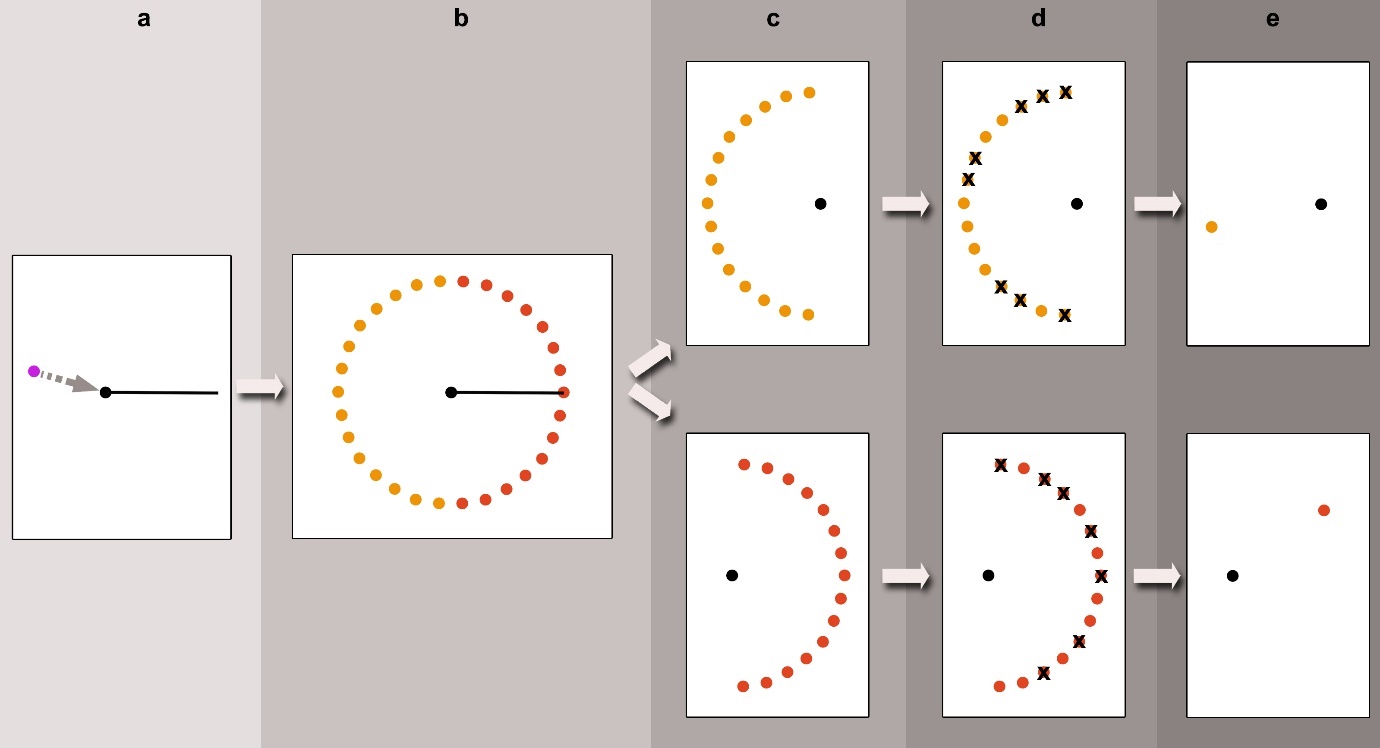


***Fig H. Simulating the position of the new movement of the second daughter cell resulted from division of the original cell.*** *(A) Location of the original cell before (magenta) and after (black) division, the black line represents the distance between the initial location of the second cell and the new location. (B) All possible locations of the new location of the second cell. (C) Possible locations in case of forward movement (red dots) or backward movement (orange dots). (D) Excluding the spots occupied by other cells. (E) Stochastic selection of the location of the the new location of the second cell in case of forward movement (red dot) or backward movement (orange dot)*

vii) Based on the lifespan length (*T*) of a cell, it can be classified as undergoing proliferation, quiescent or necrotic. If a cell is not undergoing cell division within a particular period of cell-cycles (*n_n_*) then it is considered necrotic and removed from the field (*n_n_ × d_t_ < T*). Cells that did not divide within a given period of cell-cycles (*n_s_* with *n_s_ < n_n_*) are considered as quiescent cells (*n_s_ × d_t_< T*  and  *T < n_c_ × d*_t_). Otherwise, cells are considered to undergo proliferation (*T ≤ n_s_ × d_t_*). In our model quiescent cells are mainly dependent on the local cell density. Changes in the local cell density can shift a quiescent cell back to a proliferating state or otherwise it becomes necrotic after a given period.

viii) Cells moving towards the lumina can intravasate with probability *P_i_*. The lumen can either act as an absorbing barrier, if the cell succeeded entering the lumen, or as a reflecting barrier if the cell failed entering the lumen. If a cell fails entering the lumen, it stays in its previous location. On the other hand, intravasating cells are removed from the simulation field.

Next we describe the algorithm that is used to simulate each time step. At each time (*i*), the following events (*e*) are happening per cell (*c*). Note that cell actions are typically executed at step *i+1* to account for additional time that cells may require to translate external cues into new behaviors.

*e1* -  Checking the location of the cell (*x,y* coordinates)

*e2* - Checking the proximity to a blood vessel. If the cell is located in a well-nourished zone, the cell is labeled as “nourished”.

*e3* - Checking the local cell density:

*e3.1* - If all the spots around the target simulated cell in the Moore neighborhood are occupied, then the cell will stay in place without being able to move, divide or intravasate. The cell state for quiescence and necrosis is investigated and reported.

*e3.1.1* - If the cell is necrotic, then it will be removed in the next iteration step (*i +1*). Then *e5*.

e3.1.2 - If the cell is quiescent, then it will maintain the same location in the next iteration step (*i +1*). Then *e5*.

*e3.2* - If one or more spots around the cell (including quiescent cells) in the Moore neighborhood is/are available, then *e4*.

*e4* - Checking if the cell is ready to divide by computing the proliferation score (*PS*) and checking the time spent in the well-nourished zone.

*e4.1* - If the cell does not fulfill the conditions for cell division, then *e4.1.1* and e4.1.2 respectively.

e4.1.1 - Determining the migration direction, whether it is a forward or backward movement based on the persistence ratio.

*e4.1.2* - Computing the possible new locations (*x,y* coordinates), based on the given speed, and randomly selecting a new location from the available ones. There are here three scenarios:

*e4.1.2.1* - If the cell is labeled as “nourished” and the new location is outside the nourished zone, we check if the cell should stay or leave the nourished zone (see model description, iv). In case the condition to stay in the nourished zone is fulfilled then the cell will maintain the same *x,y* coordinates in the next iteration step (*i +1*). Then *e5*.

e4.1.2.2 - If the new location is the lumen inside the nourished zone, we check if the cell will intravasate (see model description, viii). In case the condition to intravasate is fulfilled then the cell will be labeled as “intravasating cell” and removed in the next iteration step (*i +1*). Then *e5*.

e4.1.2.3 - If the new location is inside the nourished zone but not the lumen, then the cell will migrate in the next iteration step (*i +1*) to the selected new location. Then *e5*.

*e4.2* If the cell fulfills the conditions for cell division, then two new cells will appear in the next iteration step (*i +1*). The first daughter cell will maintain the same location as the mother cell whereas the location of the second daughter cell is placed stochastically based on the available spots in the Moore neighborhood around the original cell before it divided.

*e5* End of the iteration step *i* for cell *c*.

**Model initialization and parameterization**

Here we explain the choice of model parameters and initial conditions used in the simulations.

- 1. **Model initialization**

For simplicity, we start the simulations with a single cell randomly placed at location *(X_0_,Y_0_)* within the 2D simulating field *F*. The coordinates of the first movement *(X_1_,Y_1_)* of both the tumor initiating cell and the newly generated cells are stochastically computed by generating a random number (*rand*) between 0 and 1 and using it according to the following equations:

*X_1_= X_0_ + [cosine (rand × 2π) × distance]*                                        (Equation S9)

*Y_1_= Y_0_ + [sine (rand × 2π) × distance]*                                   (Equation S10)

Within the simulation field, a certain number of blood vessels (*N_V_* ) is stochastically distributed, assuming the spatial distribution of well-nourished zones surrounding blood vessels lumina is characterized by a minimum distance between any neighboring zones (*d_W_*).

- 1. **Model parameters**

A summary of all model parameters and values used is shown in Table A. Below we provide an explanation of the choices made.

Table A. A description of the parameters used in the proposed model.

| Parameter | | Description | Value | Unit | Source |
| --- | --- | --- | --- | --- | --- |
| *d* | | Cell diameter | 20 | µm | Empirical & estimated based on (1-4) |
| *F* | | Simulation field area | 16 | mm^2^ | Estimated based on ”d” |
| *L_i_* | | Surface area of the lumen | 400 | µm^2^ | Estimated based on ”d” |
| *N_V_* | | Number of blood vessels | 36 | Vessel count | Estimated based on (5) |
| *Rrows* | | A number between 1 and 5 determining the number of red boxes in the four directions around the blood vessel. The total surface area of the well-nourished zones is computed as: *W = (N_V_ × d^2^ × (2 × Rrows+1)^2^ ) - L* | 4 | Rows around blood vessels | Estimated based on (6,7) |
| *d_t_* | | Doubling time in poorly nourished zones. | 156 | Hours | Estimated based on (8) |
| *d_tw_* | | Doubling time in well*-*nourished zones around blood vessels. | 108 | Hours | Empirical & estimated based on (8-10) |
| *l* | | Low division probability | 0.0001 | Dimensionless | Assumed in this study |
| *h* | | High division probability | 0.999 | Dimensionless | Assumed in this study |
| *d_t_* | | Doubling time in poorly nourished zones. | 156 | Hours | Estimated based on (8) |
| *d_tw_* | | Doubling time in well*-*nourished zones around blood vessels. | 108 | Hours | Empirical & estimated based on (8-10) |
| *n_n_* | | Number of cell-cycle periods that leads to cell death if the division did not happen during that period. | 3 | Cell-cycle count | Estimated based on (11,12) |
| *n_s_* | | Number of cell-cycle periods that leads to cell death if the division did not happen during that period. | 2 | Cell-cycle count | Scaled based on ” n_n_” |
| *LeavingRzone* | | Probability of leaving a well*-*nourished zone. (a value between 0 and 1)  For a cell to leave the well*-*nourished zone, it should get a random number that is equal to the "LeavingRzone " or less. | 0.5 | Dimensionless | Selected based on the outcome of sensitivity analysis |
| *P_i_* | | Intravasation probability | 0.01 | Dimensionless | Estimated based on (13,14) |
| *I_GF_* | | Intravasation Increment with Golgi fragmentation (A value between 0.5 and 1.2 multiplied by 33) | 33[0.5, 1.2] | Dimensionless | Empirical |
| *t_G1_* | | Length of G1 phase | 60 | Hours | Estimated based on (8,15) |
| *GFp* | | Golgi fragmentation frequency | 0, 14, 25, 50, 65, 75 & 100 | % | Empirical |
| *P_r_* | | The persistence ratio value  (A value between 0 and 1) | Panel | Dimensionless | Empirical |
| Average Speed | minSpe | Minimum speed | Panel | µm/hour | Empirical |
|  | maxSpe | Maximum speed | Panel | µm/hour | Empirical |
|  | desired_meanSpe | Average speed | Panel | µm/hour | Empirical |
|  | desired_sdSpe | Speed standard deviation | Panel | µm/hour | Empirical |
| SimTime | | Simulation time | 2160 | Hours | Inspired by literature (12) |
| TimeInterval | | Time interval between steps | 1 | Hours | Scaled from  fmpirical data |

- Cell diameter (*d*)

Clinical and experimental data of triple negative breast cancer reported a variety in cell size ranging from 10µm to 23µm in diameter (1-4). Based on the distribution of reported mean size of MDA-MB-231 in the previous studies and in-house microscopic observation **(**Fig I**)**, we chose 20µm as the diameter of our simulated cells

- Simulation field area (*F*)

A tumor may contain many millions of cancer cells. Computational costs increase exponentially with the number of cells. For optimal use of available computational resources, we decided to simulate a cross-section (4mm × 4mm) of a tissue hosting a tumor. The maximum number of simulated cells at any time point is 40,000 cells.

- Surface area of the lumen (*L_i_*)

For simplicity, we chose the lumen to have a square shape with a surface area of the square of the cell diameter.

- Number of blood vessels (*N_V_*)

The number of blood vessels was estimated based on the reported micro-vessel density in triple negative breast cancer patients (stages i and ii) (5).

- *Rrows*

This parameter is used to construct the well*-*nourished zone and it is estimated based on efficiency of the passive mode of molecular exchange through blood vessels. The efficiency was reported to high only over small distances (< 100 µm)  (6,7).

- Doubling time in poorly nourished zones around blood vessels. *(d_t_)*

It was set based on the reporte*d* doubling time of MDA-MB-231 cells in vivo immune-deficient NSG mice (NOD.Cg-*Prkdc*^scid^ *Il2rg*^tm1Wjl^/SzJ) which is about 6 days (11).

- Doubling time in well*-*nourished zones around blood vessels. *(d_t_*_w_*)*

It was estimated based on literature [8-10] in addition to in-house experimentation, where BT549 cells were cultured in RPMI medium containing 3%, 9% or 15% serum for 24 and 48 hours then cells were trypsinized and counted **(**Fig I, panel B**)**.

- Number of needed arrested cell-cycle periods for necrosis to take place (*n_n_)*

Taking together the estimation of doubling time in poorly nourished zones around blood vessels and the necrotic temporal conditions reported in (11,12), we estimated a period of 3 cell cycles without division for necrosis to take place.

- Number of needed arrested cell-cycle periods for senescence to take place (*n_s_)*

Taking together the estimation of doubling time in poorly nourished zones around blood vessels and the estimation of (*n_n_),* we estimated a period of 2 cell cycles without division for senescence to take place.

- Probability of leaving *a* well*-*nourished zone *(LeavingRzone)*

It was set to 0.5 meaning that there is a 50% chance of accepting a movement of a cell in the well*-*nourished zone to go out. Sensitivity analysis showed that changing the value of the LeavingRzone does not change the trend of the results.

- Intravasation probability (*P_i_*)

The (*P_i_*) was estimated based on literature (13,14). Although the number of cells increases with the simulation time, the (*P_i_*) was kept constant during the simulation. Because of the minor change of intravasation rate with increased tumor size in breast cancer (16,17).

- Intravasation Increment with Golgi fragmentation (*I_GF_*)

Our experimentation showed that Golgi fragmentation increases the intravasation rate 33-fold (Fig I, panel C). In order to better mimic the empirical data, instead of using a single value, *I_GF_* can take a value within a range stochastically. This value will be used to adjust the intravasation rate:

*Intravasation Rate _(with Golgi fragmentation)_ =  P_i_× I_GF_*                           (Equation S11)

- Length of G1 phase (*t_G1_*)

The Length of G1 phase was reported to be (18–24 h) (15), by scaling it based on the total cell cycle length in vivo [8], we chose 60h as a good value for G1.

- Golgi fragmentation frequency (*GFp*)

It can take a set of values: 0, 14, 25, 50, 65, 75 & 100. Only 14 and 65 were estimated empirically. However, the other values were selected for comparison purposes.


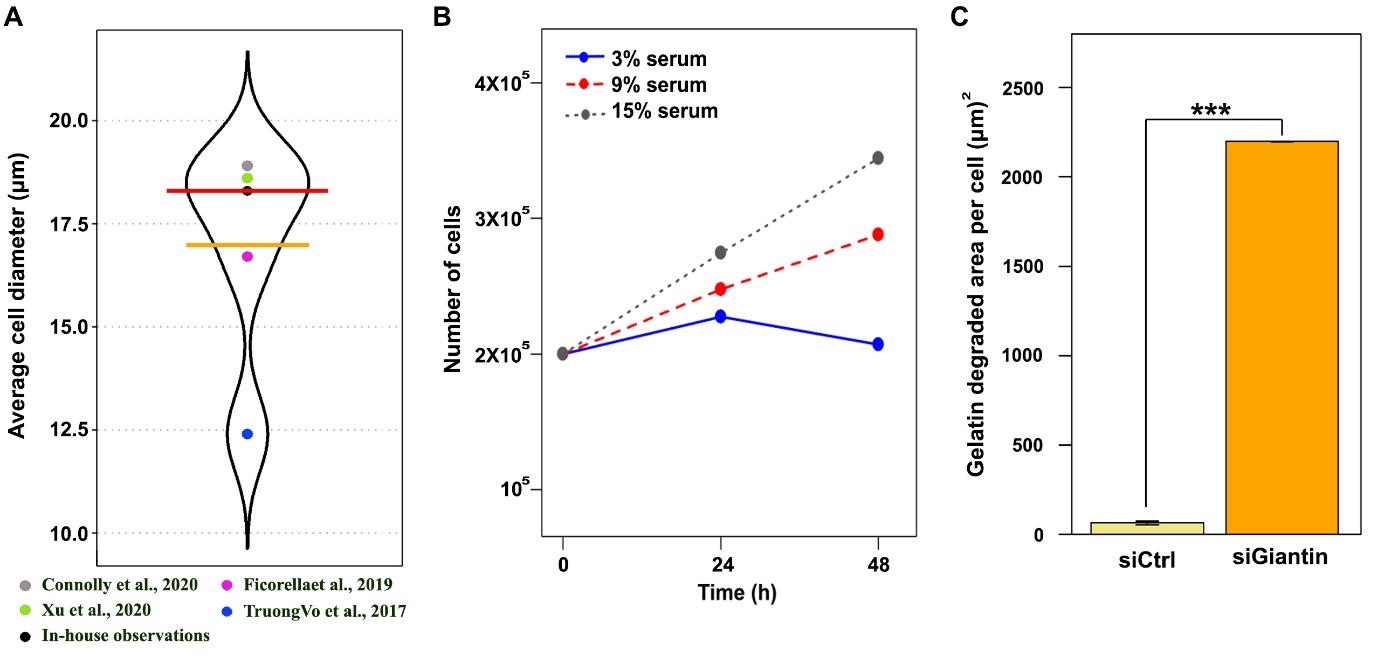


***Fig I. Experimental observations of BT548 and MDA-MB-231 cells.*** *(A) Violin plot showing of reported mean diameter of MDA-MB-231 in the several studies and in-house microscopic observation. Median cell diameter is shown in red whereas the mean is shown in orange. (B) line plot showing the number of BT549 cells* cultured in RPMI medium containing 3%, 9% or 15% serum *at three different time points. (C) Quantification of the gelatin-degraded area. Bars represent the median value. Error bars represent quantile-based coefficient of variation relative to control cells by Kruskal-Wallis chi-squared test (****p*< 0.001), n = 13 microscopic fields from 2 independent experiments.*

- The persistence ratio P_r_ and average speed

In order to mimic the movement of both MDA-MB-231 and BT549 cells, we grow them in 2D cultures by seeding 20 × 10^3^ mother cells and 5 × 10^3^ cells stably expressing fluorescent proteins that localize to the nucleus. Cells with fluorescent nuclei were tracked analyzed using our R package cellmigRation (16). In total, 1398 MDA-MB-231 cells and 2850 BT549 cells were analyzed. Based on the violin plots of the speed and persistence ratio of cells (Fig J, panels A and B), a set of 10 persistence values and 13 speeds were sampled from the distribution of empirical values to be used as input for the modeling with 130 speed-persistence combinations generated by the Cartesian product of the persistence and speed sets (Fig J, panel C). The experimental data showed a significant moderated correlation between speed and persistence ratio (r_BT549_ = 0.44, r_MDA_MB_231_ = 0.47, p-value < 0.001) (Fig J, panel D). Based on our experimental observation, we noticed that cancer cells can move from one location to another by crossing through locations that are occupied by other cells. We noticed also that cells move either in the same direction as the previous movement (Fig J, panel E) within bilateral 90 degrees (red semi-circle) or in opposite direction to the previous movement within bilateral angles between 90 and 180 degrees (orange semi-circle).

- Simulation time (SimTime)

The number of cells increases very fast with time, dramatically increasing the needed computational costs. Thus, we set the simulation time to 90 days inspired by literature (12).

- Time interval between steps (Timeinterval)

In our experimentation we used a time interval of 10min. Due to the computational cost of applying short time intervals, we decided to set the time interval to 1 hour.


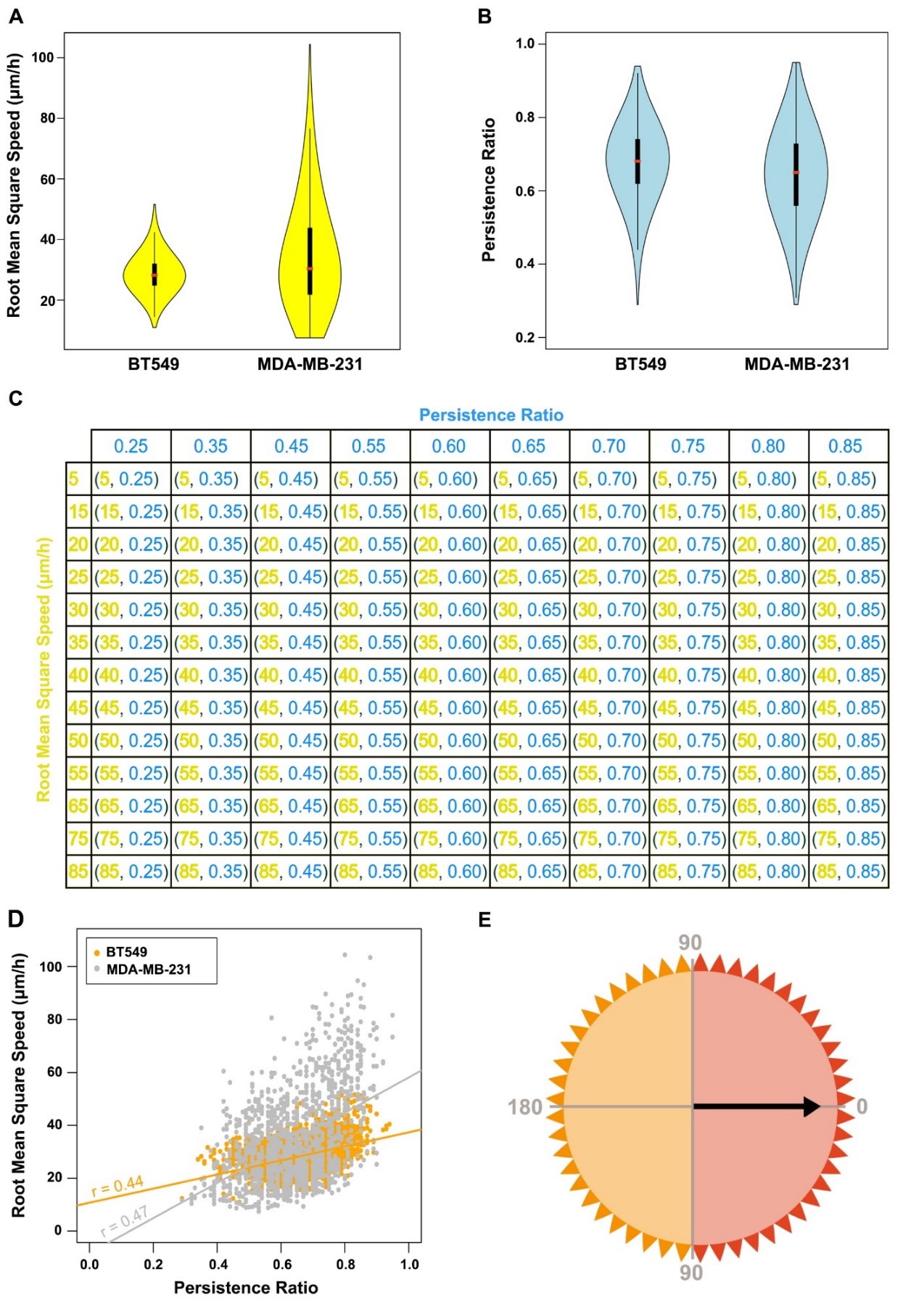


***Fig J. Experimental observations of Bt548 and MDA-MB-231 cells.*** *(A) Violin plot showing the distribution of the values of root mean square speed for BT549 and MDA_MB_231 cells. (B) Violin plot showing the distribution of the values of persistence ratio for BT549 and MDA_MB_231 cells. (C) Cartesian product of the persistence ratio and the root mean square speeds used for the modeling. (D) A scatterplot showing the relationship between the persistence ratio and the root mean square speeds of BT549 cells (orange) and MDA_MB_231 cells (gray). (E) Movement options of simulated cells. Black arrow represents the previous movement. Red triangles represent the possible locations of a forward movement whereas orange triangles represent the possible locations of a backward movement.*

### Simulation set-up and additional computational details

In order to mimic the behavior of breast cancer cells computationally, we selected simulation scenarios based on experimental observations. We constructed the proposed model to mimic the pathophysiological traits of a low-grade triple-negative breast cancer based on in-house experimentation and inspired by literature. However, the parametric structure of our model makes it applicable to different tumors simply by setting optimal values for a panel of parameters, such as the number of blood vessels, simulation time, cell speed and Golgi fragmentation frequency. In table B, we demonstrate the parameters used in the proposed model with their corresponding computational name.

In our model, the ability of a cell to move, divide, intravasate and leave a well-nourished zone is a stochastic event. Such stochasticity is achieved by generating a random number (*R∈ [0,1]*) per cell per process per iteration. A process is triggered when the random number is equal to or less than the corresponding parameter value. For example, to compute the direction of movement, a random number between 0 and 1 is generated. In case the random number is equal or greater than the *P_r_*, then the cell will move in the opposite direction to the previous movement. However, if the random number is less than the *P_r_*, then the cell will move in the same direction as the previous movement. To achieve this, we assume that the new location is one point from all the points on the circle circumference with a radius of a particular distance computed from the speed profile (Fig F, panel A). To get these points, first a series of trigonometry equations (Fig K, panel A) are used to compute the coordinates (*x_3_,y_3_*) of the outermost point of the radius (*p_3_*) that forms a right angle with the distance of the previous movement (D_1_). Then, the coordinates of the points on the circle circumference are computed using the following equations:

$X_{i}=x_{2}+cos(i \times\pi/180) \times(x_{3}-x_{2})-sin(i\times\pi/180)\times(y_{3}-y_{2})$ (Equation S12)

$Y_{i}=y_{2}+cos(i \times\pi/180) \times(y_{3}-y_{2})-sin(i\times\pi/180)\times(x_{3}-x_{2})$ (Equation S13)

Where i is a set of angles. In case of forward migration, the set contains 44 angles between 184° and 356° to keep the direction of the movement within bilateral 90 degrees, whereas in backward migration the set contains 46 angles between -180° and -360° (Fig K, panel B). Finally, the grid-based spatial reference is used to select one point stochastically among the available points that are not occupied by other cells (Fig K, panel C). In case all the points are occupied, the cell will stay in place.


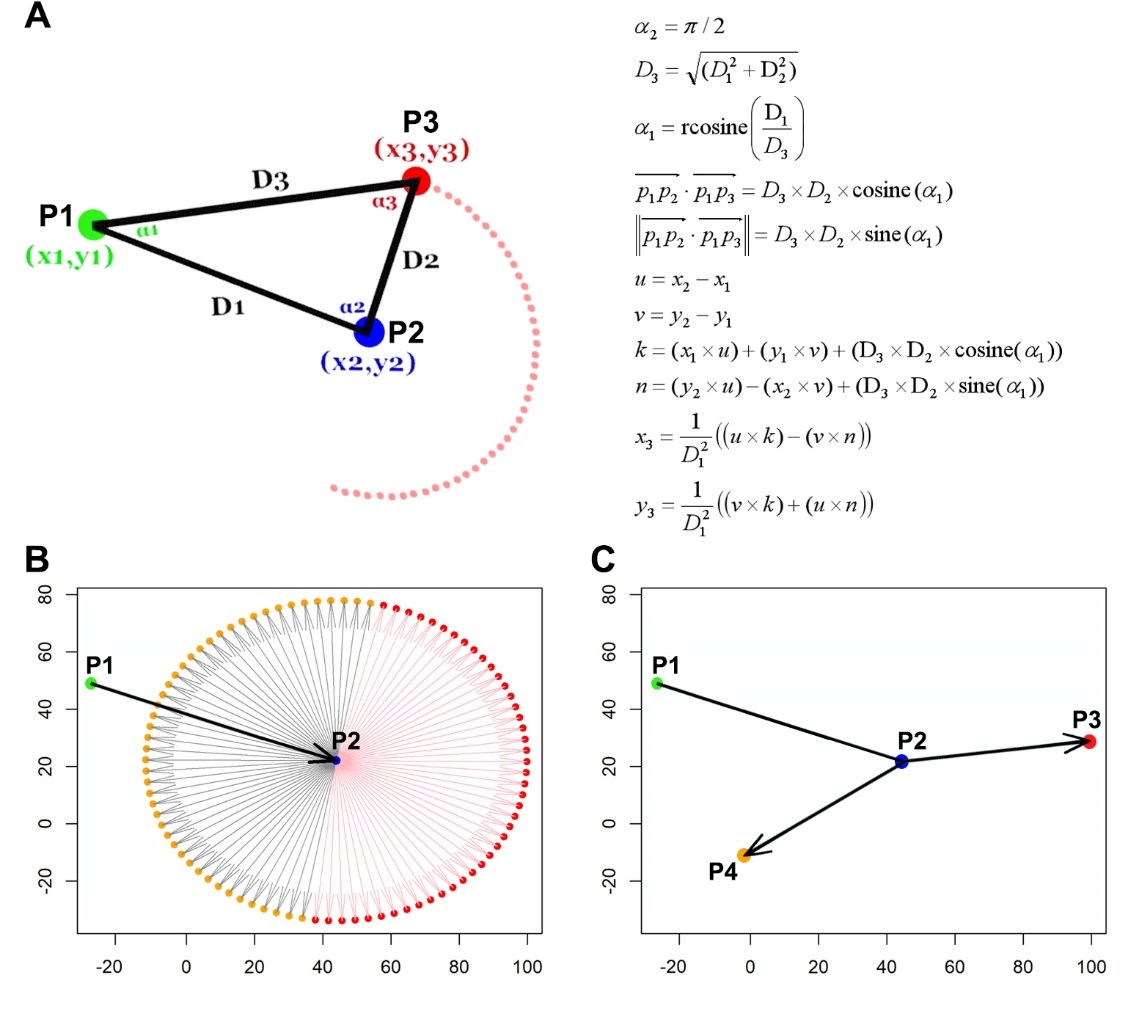


***Fig K. Simulating cell movements. (****A) A schema with a panel of mathematical equations explaining how the X and Y coordinates of the new movement are computed. (B) Possible locations in case of forward movement (red dots) or backward movement (orange points). (C) A stochastic selection of a forward movement from P2 to P3 and a backward movement from P2 to P4.*

For navigation purposes, a grid-based spatial reference system was embedded on top of the 2D simulation field. The number of grid squares in each direction of the computational grid is determined by dividing the length of the simulation field side by the diameter of the simulated cancer cell generating a grid of 40,000 squares. Each grid square is 20µm × 20µm. This lattice grid simply helps determine the cell local density around a target cell using the Moore neighborhood method *(*Fig L, panel A*).* Since a small spatial field of the tumor is simulated, periodic boundary conditions were imposed to minimize the edge effect and avoid losing cells (19). For example, if a cell migrates past the left edge, it will then appear at the right edge (Fig L, panel B). Lattice squares are numbered from 1 to 40,000, where 1 is located at the lower left corner whereas 40,000 is located at the upper right corner (Fig L, panel C). The lattice square numbering increases from left to right and bottom to top.

To evaluate the distribution of the simulated cancer cells, the uniformity index (*UI*) is computed as the average of 16 sectional uniformity indices (*UI_i_*) representing the 16 sections of the simulated field (Fig L, panel D).

$UI =\frac{1}{16}\Sigma_{i=1..16}UI_{i}$ (Equation S14)

Each sectional uniformity index (*UI_i_*) is computed as one minus coefficient of variation (*CV*) multiplied by hundred. The *CV* represents the ratio of the standard deviation to the mean of the occupied grid squares within the section.

$UI_{i}=100 \times(1-CV)$ (Equation S15)


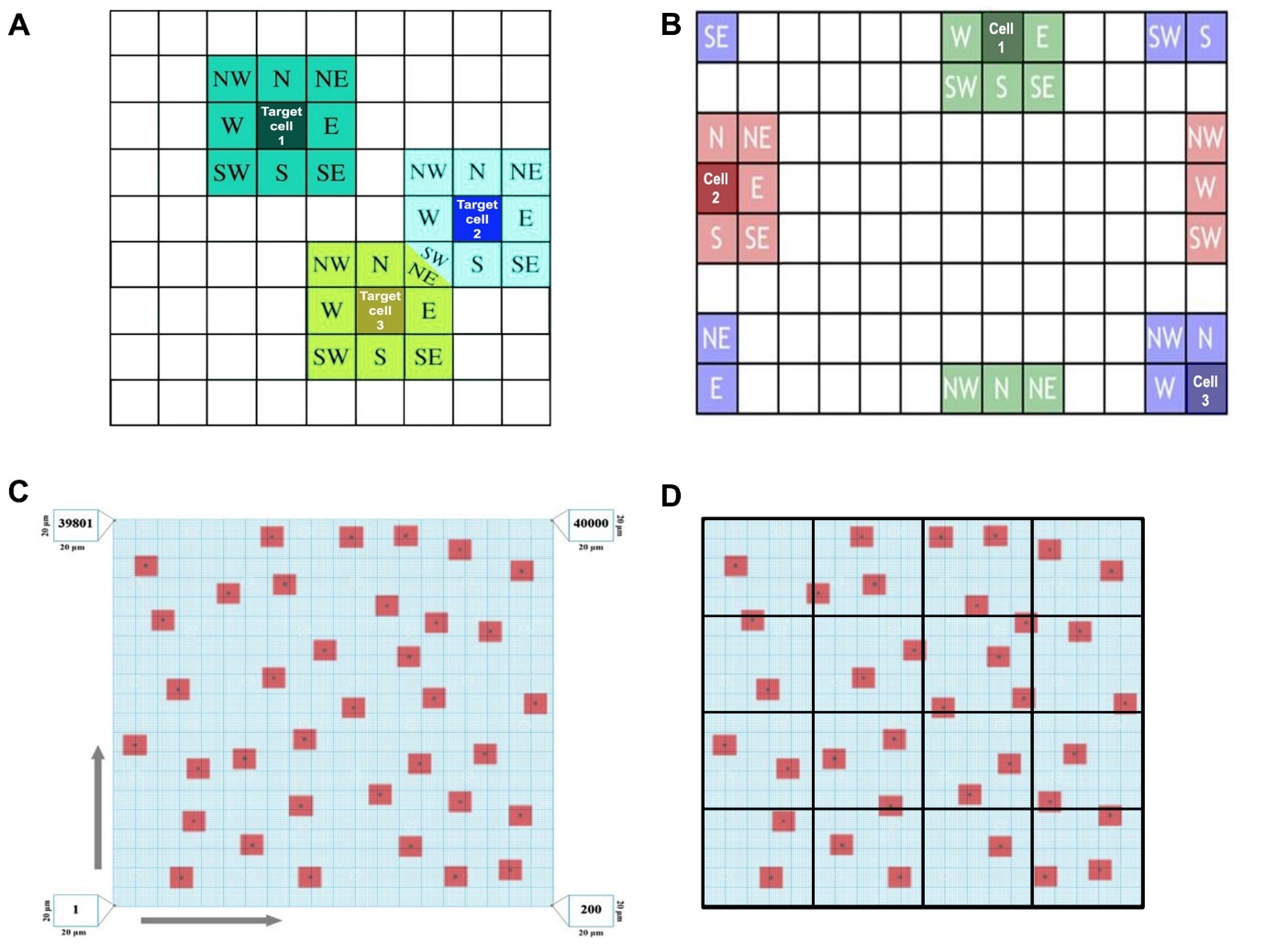


***Fig L. The simulation field*.** (A) Moore neighborhood. (B) Moore neighborhood in periodic boundary conditions. *(C) The grid-based spatial reference system embedded on top of the 2D simulation field, arrows show the increase in the lattice square numbering.*

Computer simulations were performed mainly in HPC solutions provided by the Oslo Centre for Epidemiology and Biostatistics, at the University of Oslo and also on the Abel computer cluster, owned by the University of Oslo, and operated by the Department for Research Computing at USIT, the University of Oslo IT-department. The source code of the proposed model is available at <https://github.com/ocbe-uio/Cancer_simulator>.

**Table B** Parameters used in the proposed model with their corresponding computational name.

| Parameter name | Corresponding computational name |
| --- | --- |
| *d* | _ |
| *F* | _ |
| *L_i_* | _ |
| *N_V_* | BloVesN |
| *Rrows* | Rrows |
| *d_t_* | DT |
| *d_tw_* | DTinR |
| *n_n_* | PeriodForDeath |
| *n_s_* | PeriodForSenescence |
| *LeavingRzone* | LeavingRzone |
| Intravasation probability (*P_i_*) | IntravasationProb |
| Intravasation Increment with Golgi fragmentation (*I_GF_*) | IntraVas_inc_By_GF |
| *t_G1_* | G1 |
| *GFp* | Golgi_Fra_Frequency |
| *P_r_* | PER |
| minSpe | minSpe |
| maxSpe | maxSpe |
| desired_meanSpe | desired_meanSpe |
| desired_sdSpe | desired_sdSpe |
| SimTime | SimTime |
| TimeInterval | TimeInterval |

**References**

1. Connolly S, McGourty K, Newport D. The in vitro inertial positions and viability of cells in suspension under different in vivo flow conditions. Scientific Reports **2020**;10:1-13

2. Ficorella C, Vázquez RM, Heine P, Lepera E, Cao J, Warmt E*, et al.* Normal epithelial and triple-negative breast cancer cells show the same invasion potential in rigid spatial confinement. New Journal of Physics **2019**;21:083016

3. Xu J, Jiang X, Li H, Arlinghaus LR, McKinley ET, Devan SP*, et al.* Magnetic resonance imaging of mean cell size in human breast tumors. Magnetic resonance in medicine **2020**;83:2002-14

4. TruongVo T, Kennedy R, Chen H, Chen A, Berndt A, Agarwal M*, et al.* Microfluidic channel for characterizing normal and breast cancer cells. Journal of Micromechanics and Microengineering **2017**;27:035017

5. Kraby MR, Opdahl S, Akslen LA, Bofin AM. Quantifying tumour vascularity in non-luminal breast cancers. Journal of Clinical Pathology **2017**;70:766-74

6. Carmeliet P, Jain RK. Angiogenesis in cancer and other diseases. nature **2000**;407:249-57

7. Pittman RN. The circulatory system and oxygen transport. Regulation of Tissue Oxygenation: Morgan & Claypool Life Sciences; 2011.

8. Zibara K, Awada Z, Dib L, El-Saghir J, Al-Ghadban S, Ibrik A*, et al.* Anti-angiogenesis therapy and gap junction inhibition reduce MDA-MB-231 breast cancer cell invasion and metastasis in vitro and in vivo. Scientific reports **2015**;5:1-16

9. Okumura M, Yamamoto M, Sakuma H, Kojima T, Maruyama T, Jamali M*, et al.* Leptin and high glucose stimulate cell proliferation in MCF-7 human breast cancer cells: reciprocal involvement of PKC-α and PPAR expression. Biochimica et Biophysica Acta (BBA)-Molecular Cell Research **2002**;1592:107-16

10. Fang M, Shen Z, Huang S, Zhao L, Chen S, Mak TW*, et al.* The ER UDPase ENTPD5 promotes protein N-glycosylation, the Warburg effect, and proliferation in the PTEN pathway. Cell **2010**;143:711-24

11. Wcisło R, Dzwinel W, Yuen DA, Dudek AZ. A 3-D model of tumor progression based on complex automata driven by particle dynamics. Journal of molecular modeling **2009**;15:1517-39

12. Kłusek A, Dzwinel W, Dudek AZ. Simulation of tumor necrosis in primary melanoma. 2016. p 1-8.

13. Butler TP, Gullino PM. Quantitation of cell shedding into efferent blood of mammary adenocarcinoma. Cancer research **1975**;35:512-6

14. Bockhorn M, Jain RK, Munn LL. Active versus passive mechanisms in metastasis: do cancer cells crawl into vessels, or are they pushed? The lancet oncology **2007**;8:444-8

15. Bayarmagnai B, Perrin L, Esmaeili Pourfarhangi K, Graña X, Tüzel E, Gligorijevic B. Invadopodia-mediated ECM degradation is enhanced in the G1 phase of the cell cycle. Journal of cell science **2019**;132:jcs227116

16. Braun S, Vogl FD, Naume B, Janni W, Osborne MP, Coombes RC*, et al.* A pooled analysis of bone marrow micrometastasis in breast cancer. New England journal of medicine **2005**;353:793-802

17. Hüsemann Y, Geigl JB, Schubert F, Musiani P, Meyer M, Burghart E*, et al.* Systemic spread is an early step in breast cancer. Cancer cell **2008**;13:58-68

18. Ghannoum S FD, Leoncio W, Sørensen Ø cellmigRation: Track Cells, Analyze Cell Trajectories and Compute Migration Statistics. R package version 1.2.0,. Bioconductor **2021**

19. Nolan D, Lally C. Coupled finite element–agent-based models for the simulation of vascular growth and remodeling. Numerical methods and advanced simulation in biomechanics and biological processes: Elsevier; 2018. p 283-300.

**List of Supplementary Figure Legends**

***Fig A. Impact of cell speed and persistence on tumor progression.*** *(A) Strong negative correlation between number of necrotic cells and uniformity index (Pearson correlation coefficient = -0.76). (B)  Weak negative correlation between number of* quiescent *cells and uniformity index (Pearson correlation coefficient = -0.24). (C) Number of cancer cells after 90 days from the tumor initiation across different speed-persistence combinations. (D) Number of cancer cells at different time points (blue: day 70, yellow: day 80 and gray: day 90) across gradual persistence value but fixed speed of 30um/h. (E) Number of intravasating cells after 90 days from the tumor initiation across different speed-persistence combinations. E*rror bars represent *the* standard deviation *of twenty five replicates.*

***Fig B. Immunofluorescence staining for Golgi marker giantin (yellow).*** *(A) Representative image of adjacent normal breast tissue patient samples. (B) Representative image of invasive ductal carcinoma patient section.*

***Fig C. Kaplan–Meier survival curves*** *for* six matrix proteins that have a relatively well understood role in maintaining Golgi morphology (*GOLGA1, GOLGA2, GOLGA3, GRASP55, GOLGB1* and *GORASP1*).

***Fig D. Impact of Golgi fragmentation on cell migration.*** *(A) Percentage of cells denoted with fragmented Golgi in BT549 cells. (B) Bar plot showing the percentage of cells based on speed, low speed includes group 1&2 whereas high speed includes group 3&4 . (C) Bar plot showing the percentage of cells based on persistence, low persistence includes group 1&3 whereas high persistence includes group 2&4. (D) Bar plot showing the averaged persistence ratio per group*

***Fig E. Number of intravasating cells in a population of a thousand cancer cells.*** *Statistical significance was determined using the Kruskal–Wallis rank sum test, followed by pairwise comparisons using the Wilcoxon rank sum test with* Bonferroni Correction *(* p < 0.05, *** p < 0.001).*

***Fig F. Cell’s speed-persistence.*** *(A) 5-day speed profile. (B) Schematic chart explaining how the persistence ratio is computed for a cell that moved from a to b.*

***Fig G. Simulation field and the local density.*** *(A) A 2D section showing the architecture of the simulated tumor field. Black squares represent the lumen of blood vessels. Red squares represent the well-nourished zones. The blue circle represents a single cancer cell. (B) Schema explaining the way of computing the local cell density.*

***Fig H. Simulating the position of the new movement of the second daughter cell resulted from division of the original cell.*** *(A) Location of the original cell before (magenta) and after (black) division, the black line represents the distance between the initial location of the second cell and the new location. (B) All possible locations of the new location of the second cell. (C) Possible locations in case of forward movement (red dots) or backward movement (orange dots). (D) Excluding the spots occupied by other cells. (E) Stochastic selection of the location of the the new location of the second cell in case of forward movement (red dot) or backward movement (orange dot)*

***Fig I. Experimental observations of BT548 and MDA-MB-231 cells.*** *(A) Violin plot showing of reported mean diameter of MDA-MB-231 in the several studies and in-house microscopic observation. Median cell diameter is shown in red whereas the mean is shown in orange. (B) line plot showing the number of BT549 cells* cultured in RPMI medium containing 3%, 9% or 15% serum *at three different time points. (C) Quantification of the gelatin-degraded area. Bars represent the median value. Error bars represent quantile-based coefficient of variation relative to control cells by Kruskal-Wallis chi-squared test (****p*< 0.001), n = 13 microscopic fields from 2 independent experiments.*

***Fig J . Experimental observations of Bt548 and MDA-MB-231 cells.*** *(A) Violin plot showing the distribution of the values of root mean square speed for BT549 and MDA_MB_231 cells. (B) Violin plot showing the distribution of the values of persistence ratio for BT549 and MDA_MB_231 cells. (C) Cartesian product of the persistence ratio and the root mean square speeds used for the modeling. (D) A scatterplot showing the relationship between the persistence ratio and the root mean square speeds of BT549 cells (orange) and MDA_MB_231 cells (gray). (E) Movement options of simulated cells. Black arrow represents the previous movement. Red triangles represent the possible locations of a forward movement whereas orange triangles represent the possible locations of a backward movement.*

***Fig K. Simulating cell movements. (****A) A schema with a panel of mathematical equations explaining how the X and Y coordinates of the new movement are computed. (B) Possible locations in case of forward movement (red dots) or backward movement (orange points). (C) A stochastic selection of a forward movement from P2 to P3 and a backward movement from P2 to P4.*

***Fig L. The simulation field*.** (A) Moore neighborhood. (B) Moore neighborhood in periodic boundary conditions. *(C) The grid-based spatial reference system embedded on top of the 2D simulation field, arrows show the increase in the lattice square numbering.*

**List of Supplementary Table Legends**

***Table A.*** A description of the parameters used in the proposed model.

***Table B.*** Parameters used in the proposed model with their corresponding computational name.
